# Development and validation of an interpretable ensemble model for predicting androgen receptor status in triple-negative breast cancer: a multi-center study

**DOI:** 10.3389/fonc.2026.1743315

**Published:** 2026-03-11

**Authors:** Mei Ruan, Lixiu Cao, Yongliang Liu, Yanna Shan, Zhi Li, Chang Shao, Wen Xu

**Affiliations:** 1 Department of Radiology, Affiliated Hangzhou First People’s Hospital, School of Medicine, Westlake University, Hangzhou, China; 2 Department of Nuclear Medicine Imaging, Tangshan People’s Hospital, Tangshan, China; 3 Department of Medical Services, Tangshan People’s Hospital, Tangshan, China; 4 Department of Radiology, The First Affiliated Hospital, School of Medicine, Zhejiang University, Hangzhou, China; 5 Department of Pathology, Affiliated Hangzhou First People’s Hospital, School of Medicine, Westlake University, Hangzhou, China

**Keywords:** androgen receptor, ensemble learning, multi-parameter MRI, radiomics, triple-negative breast cancer

## Abstract

**Purpose:**

Reliable assessment of androgen receptor (AR) status in triple-negative breast cancer (TNBC) is critical for targeted therapy but remains challenging due to biopsy limitations from intratumoral heterogeneity. This study aimed to develop and validate an interpretable ensemble model integrating radiomics and multiparametric MRI for noninvasive AR status prediction.

**Materials and methods:**

A total of 379 TNBC patients from three institutions were included for model training and external validation. All patients underwent preoperative dynamic contrast-enhanced MRI. Radiomic features were extracted from a Segment Anything Model-based segmentation tool and underwent multi-step selection. Multiparametric MRI features were evaluated using standardized criteria. Three predictive models, including a radiomics model, an MRI model, and an integrated ensemble model, were constructed using a stacking framework with Random Forest, XGBoost, and LightGBM. Model performance was assessed by ROC analysis, calibration, and decision curve analysis. SHapley Additive exPlanations (SHAP) were applied for interpretability.

**Results:**

The integrated model achieved the best performance (AUC = 0.891 in the training cohort), outperforming radiomics (AUC = 0.836) and MRI models (AUC = 0.753). External validation confirmed robustness (AUC = 0.863 and 0.818). The integrated model maintained high sensitivity (78–85%) and specificity (82–87%) across cohorts. SHAP analysis revealed radiomic descriptors, especially skewness and surface-to-volume ratio, as the most influential predictors.

**Conclusions:**

An interpretable ensemble model integrating radiomics and multiparametric MRI achieved robust and generalizable performance for AR status prediction in TNBC. This noninvasive approach may assist in patient stratification for AR-targeted therapy and support personalized treatment strategies.

## Introduction

Triple-negative breast cancer (TNBC), characterized by the absence of estrogen receptor (ER), progesterone receptor (PR), and HER2 expression, accounts for 15–20% of all breast cancers and is associated with aggressive behavior, early relapse, and poor prognosis (1, 2). Breast cancer remains a major global health burden; GLOBOCAN 2022 estimated approximately 2.30 million new cases and 666,000 deaths worldwide in 2022 (3). Unlike hormone receptor–positive or HER2-amplified subtypes, effective targeted therapies for TNBC remain limited for many patients, and chemotherapy continues to play a central role in routine management (4). Meanwhile, antibody–drug conjugates and other emerging systemic options for HER2-negative disease are reshaping treatment strategies and further emphasize the need for clinically meaningful biomarkers to guide patient selection (5). This highlights an urgent clinical need for novel biomarkers to improve patient stratification and guide personalized treatment strategies (6, 7).

The androgen receptor (AR) has emerged as a clinically relevant biomarker and potential therapeutic target in TNBC. Reported AR positivity in TNBC varies substantially across studies (approximately 10–50%), largely due to differences in immunohistochemistry assays and cut-off definitions (commonly ≥1% vs ≥10%), as well as cohort composition (8). The prognostic and predictive implications of AR positivity in TNBC have also been inconsistently reported, with some studies suggesting inferior response to chemotherapy or worse outcomes, while others report no clear prognostic association (9, 10). Clinically, phase II trials of AR-targeted agents have shown modest but measurable activity in selected AR-positive TNBC: bicalutamide achieved a 6-month clinical benefit rate (CBR) of 19% (11), enzalutamide reported a 16-week CBR of 25% in the intent-to-treat population (33% in the evaluable subgroup) (12), and abiraterone acetate plus prednisone reported a 6-month CBR of 20% (13). Accurate identification of AR status is therefore critical for treatment planning. However, AR assessment is currently biopsy-based, and intratumoral heterogeneity often leads to discrepancies between biopsy and surgical specimens, with concordance rates ranging widely from 47.8% to 97.8% (8–12). This limitation challenges reliable patient selection for AR-targeted therapy.

Noninvasive imaging provides an opportunity to capture whole-tumor biology beyond focal sampling. Multiparametric MRI has been widely used in breast cancer for evaluating morphology, enhancement kinetics, diffusion properties, and vascular physiology, offering quantitative insights into tumor heterogeneity (14, 15). Radiomics further enables high-throughput extraction of quantitative imaging features that may correlate with receptor status and molecular subtypes. Prior studies have suggested associations between MRI-derived features and AR expression, but most have been limited by single-center design, lack of external validation, or insufficient interpretability of predictive models (16, 17).

Therefore, this study aimed to develop and externally validate an interpretable ensemble model that integrates radiomics and multiparametric MRI features for noninvasive prediction of AR status in TNBC. By combining predictive performance with transparent feature attribution, this approach may support patient selection for AR-targeted therapy and contribute to more individualized treatment decision-making in clinical practice.

## Materials and methods

### Study population and design

This retrospective study received ethical approval from medical ethics committee of all three institutions. Informed consent was obtained from each participant included in the study. Between January 2015 and October 2024, we retrieved 379 breast cancer patients with surgically confirmed TNBC who underwent preoperative breast MRI at three institutions. The eligibility criteria were as follows: (i) Female patients; (ii) Pathologically confirmed TNBC with complete immunohistochemical analysis of ER, PR, HER2, and AR expression status available; (iii) Underwent DCE-MRI within two weeks prior to surgery; (iv) Imaging quality meets the requirements for radiomics analysis. The exclusion criteria were as follows: (i) Incomplete clinical or pathological data; (ii) Received neoadjuvant chemotherapy prior to MRI examination; (iii) Presence of concurrent malignant tumors; (iv) Pregnancy or lactation at the time of study inclusion.

### Pathological analysis

All surgical specimens and core biopsies were reviewed by experienced breast pathologists according to the American Society of Clinical Oncology/College of American Pathologists (ASCO/CAP) guidelines. ER, PR, and HER2 status were evaluated to confirm triple-negative phenotype. AR expression was assessed by immunohistochemistry using monoclonal anti-AR antibody. Nuclear staining in ≥10% of tumor cells was defined as AR-positive, while <10% was considered AR-negative. Histological grade, lymphovascular invasion, and nodal status were also recorded from pathology reports.

### MRI acquisition and feature assessment

All examinations were performed on 3.0-T scanners across the three institutions (Institutions I–II: Siemens Healthineers MAGNETOM Verio; Institution III: Philips Healthcare Ingenia), and the same DCE-MRI protocol framework was applied (one pre-contrast plus five post-contrast phases). A gadolinium-based contrast agent was administered using a consistent injection protocol across centers (0.2 mmol/kg at 2.0 mL/s, followed by a 20 mL saline flush; images acquired at ~1–5 minutes post-injection). Pharmacokinetic parameters (Ktrans, Ve, Kep) were derived using the Extended Tofts model with individual arterial input functions. Two radiologists (3 and 8 years of experience), blinded to pathological results, independently evaluated multi-parameter MRI features following BI-RADS lexicon, including morphological characteristics (size, shape, margin), signal intensity on T2WI/DWI, ADC values, enhancement patterns, and time-intensity curves, with a third radiologist (12 years of experience) adjudicating disagreements. Features showing significant univariate association with AR status (*p* < 0.05) were entered into multivariate logistic regression with backward stepwise selection. Random forest analysis was performed to calculate feature importance scores. Detailed acquisition parameters and assessment criteria are provided in **Supplementary Material** I-II.

### Tumor segmentation and validation

Tumor segmentation was conducted using MedSAM-Lite integrated with 3D Slicer (version 5.6.2), a Segment Anything Model–based tool fine-tuned for medical imaging. Radiologists drew bounding boxes around the tumor on T2WI and the second post-contrast DCE-MRI phase, after which the algorithm generated tumor masks automatically. Minimal manual refinement was permitted when necessary. To validate this approach, 50 randomly selected cases were independently segmented by two radiologists (3 and 8 years of experience) using both manual and MedSAM-assisted methods, with a 7-day interval to minimize recall bias. Manual segmentations served as the reference standard. Segmentation accuracy and efficiency were evaluated.

All automated masks underwent quality control by a senior radiologist (12 years of experience) blinded to pathological results. Cases requiring >20% manual correction were excluded. The final protocol demonstrated excellent agreement with manual segmentation (**Supplementary Tables S1**, **S2**) and significantly reducing average segmentation time from 15.3 ± 3.2 minutes to 2.8 ± 0.7 minutes (*p* < 0.001).

### Radiomic feature extraction and selection

All images underwent standardized preprocessing, including voxel size resampling (1 × 1 × 1 mm³), intensity normalization, and scanner harmonization. Radiomic feature extraction was performed on the uAI Research Portal platform, yielding 1888 features encompassing first-order statistics, shape, and multiple texture categories (GLCM, GLRLM, GLSZM, NGTDM). A multi-step selection strategy was applied to ensure reproducibility and relevance. Features with intraclass correlation coefficients (ICC) < 0.75 were excluded. Stability was then assessed through bootstrap resampling (1000 iterations), and highly correlated features (|r| > 0.90) were removed. Minimum redundancy–maximum relevance (mRMR) was applied to optimize feature selection, followed by the Boruta algorithm to identify variables with significant discriminative value. The retained features were tested using the Mann–Whitney U test with Benjamini–Hochberg correction for multiple comparisons (*p* < 0.05). Further details are provided in **Supplementary Material** III.

### Model development

Three predictive models were constructed using a stacking ensemble approach: a radiomics model, a multiparametric MRI model, and an integrated model combining both feature sets. The ensemble consisted of three base learners (Random Forest, XGBoost, and LightGBM) with optimized regularization parameters, and a logistic regression meta-learner. Model training was performed with nested five-fold cross-validation, where base learners generated meta-features in the inner loop for training the meta-learner in the outer loop (see **Supplementary Material** III for details).

### Model interpretation and statistical analysis

This framework applies cooperative game theory to quantify the contribution of each feature to individual predictions. SHAP Kernel Explainer was used to compute marginal feature contributions while accounting for feature interactions. Visualization included summary plots, illustrating overall feature importance and distribution, and force plots, providing case-level explanations for individual patients. Model performance was evaluated using ROC analysis with AUC calculation, DeLong test for comparisons, sensitivity, specificity, and accuracy. Calibration was assessed using Hosmer-Lemeshow test. Decision curve analysis evaluated clinical utility. Continuous variables were compared using t-tests or Mann-Whitney U tests, categorical variables using chi-square tests. Multiple testing correction employed Benjamini-Hochberg method. Statistical significance was set at *p* < 0.05. Analysis was performed using Python 3.11.3 and SPSS V.25.0.

## Results

### Patient characteristics

The primary training cohort from institution I included 210 TNBC patients, comprising 146 (69.5%) AR-negative and 64 (30.5%) AR-positive cases. Two external validation cohorts were analyzed: the TS cohort (n = 112; 80 [71.4%] AR-negative, 32 [28.6%] AR-positive) and the ZJU cohort (n = 57; 41 [71.9%] AR-negative, 16 [28.1%] AR-positive). The mean age did not differ significantly between AR-positive and AR-negative patients (54.7 ± 12.1 vs 52.3 ± 11.4 years, *p* = 0.283). Other clinicopathological characteristics—including menopausal status, Ki-67 expression, T stage, N stage, lymphovascular invasion, and histological type—also showed no significant group differences (all *p* > 0.05). Baseline features were comparable across the three cohorts (all *p* > 0.05), supporting the validity of external validation (**Tables 1**, **2**).

**Table 1:** Baseline characteristics of patients in different cohorts.

Characteristics	Primary Cohort (n=210)	TS cohort (n=112)	ZJU cohort (n=57)	*P* value
Age (years), mean ± SD	52.9 ± 11.6	53.4 ± 12.2	51.8 ± 10.9	0.693
Menopausal status, n (%)				0.876
Premenopausal	112 (53.3)	58 (51.8)	32 (56.1)	
Postmenopausal	98 (46.7)	54 (48.2)	25 (43.9)	
Ki-67, n (%)				0.824
≤ 20% (low)	45 (21.4)	26 (23.2)	11 (19.3)	
> 20% (high)	165 (78.6)	86 (76.8)	46 (80.7)	
T stage, n (%)				0.997
T1	78 (37.1)	43 (38.4)	19 (33.3)	
T2	98 (46.7)	52 (46.4)	29 (50.9)	
T3 or T4	34 (16.2)	17 (15.2)	9 (15.8)	
N stage, n (%)				0.998
N0	126 (60.0)	69 (61.6)	32 (56.1)	
N1	56 (26.7)	28 (25.0)	17 (29.8)	
N2 or N3	28 (13.3)	15 (13.4)	8 (14.0)	
Lymphovascular invasion, n (%)				0.824
Absent	143 (68.1)	78 (69.6)	37 (64.9)	
Present	67 (31.9)	34 (30.4)	20 (35.1)	
Histological type, n (%)				0.943
Invasive ductal carcinoma	189 (90.0)	102 (91.1)	50 (87.7)	
Invasive lobular carcinoma	12 (5.7)	6 (5.4)	4 (7.0)	
Others	9 (4.3)	4 (3.6)	3 (5.3)	

**Table 2:** Comparison of baseline characteristics between AR-positive and AR-negative patients.

Characteristics	AR-positive (n=112)	AR-negative (n=267)	*P* value
Age (years), mean ± SD	54.7 ± 12.1	52.3 ± 11.4	0.283
Menopausal status, n (%)			0.346
Premenopausal	57 (50.9)	145 (54.3)	
Postmenopausal	55 (49.1)	122 (45.7)	
Ki-67, n (%)			0.412
≤ 20% (low)	28 (25.0)	54 (20.2)	
> 20% (high)	84 (75.0)	213 (79.8)	
T stage, n (%)			0.487
T1	39 (34.8)	101 (37.8)	
T2	54 (48.2)	125 (46.8)	
T3 or T4	19 (17.0)	41 (15.4)	
N stage, n (%)			0.563
N0	65 (58.0)	162 (60.7)	
N1	31 (27.7)	70 (26.2)	
N2 or N3	16 (14.3)	35 (13.1)	
Lymphovascular invasion, n (%)			0.298
Absent	73 (65.2)	185 (69.3)	
Present	39 (34.8)	82 (30.7)	
Histological type, n (%)			0.456
Invasive ductal carcinoma	99 (88.4)	242 (90.6)	
Invasive lobular carcinoma	8 (7.1)	14 (5.2)	
Others	5 (4.5)	11 (4.1)	

### Multi-parameter MRI features analysis

Interobserver agreement in MRI feature assessment was good to excellent (κ = 0.82–0.91). Univariate analysis identified five features significantly associated with AR status. Among pharmacokinetic parameters, AR-positive tumors demonstrated higher Ktrans [(0.38 ± 0.14) min^-^¹ vs (0.31 ± 0.13) min^-^¹, *p* = 0.024], Ve [(0.26 ± 0.09) vs (0.22 ± 0.08), *p* = 0.015], and Kep [(1.46 ± 0.52) min^-^¹ vs (1.41 ± 0.50) min^-^¹, *p* = 0.042] values compared with AR-negative tumors. ADC values were also lower in AR-positive tumors [(0.97 ± 0.18) × 10^-^³ mm²/s vs (0.99 ± 0.22) × 10^-^³ mm²/s, *p* = 0.044], and TIC patterns were significantly associated with AR status (*p* = 0.047). Other morphological and enhancement features did not differ between groups (**Table 3**).

**Table 3:** Comparison of MRI features between AR-positive and AR-negative TNBC in the training cohort.

MRI features	AR-positive (n=64)	AR-negative (n=146)	*P* value
Maximum diameter (cm), mean ± SD	3.2 ± 1.1	3.0 ± 1.0	0.186
ADC value (×10^-^³ mm²/s), mean ± SD	0.97 ± 0.18	0.99 ± 0.22	0.044
Tumor shape, n (%)			0.068
Round/oval	14 (21.9)	48 (32.9)	
Irregular	42 (65.6)	85 (58.2)	
Lobular	1 (1.6)	1 (0.7)	
Nonmass enhancement	7 (10.9)	12 (8.2)	
Background parenchymal enhancement, n (%)			0.079
None or minimal	15 (23.4)	42 (28.8)	
Mild	25 (39.1)	59 (40.4)	
Moderate	19 (29.7)	35 (24.0)	
Marked	5 (7.8)	10 (6.8)	
Margin, n (%)			0.087
Circumscribed	6 (9.4)	25 (17.1)	
Irregular	48 (75.0)	103 (70.5)	
Spiculated	10 (15.6)	18 (12.4)	
Peritumoral edema, n (%)			0.054
Present	38 (59.4)	69 (47.3)	
Absent	26 (40.6)	77 (52.7)	
Enhancement pattern, n (%)			0.127
Homogeneous	9 (14.1)	33 (22.6)	
Heterogeneous	55 (85.9)	113 (77.4)	
T2WI signal intensity, n (%)			0.219
Hyperintense	22 (34.4)	59 (40.4)	
Isointense	37 (57.8)	74 (50.7)	
Hypointense	5 (7.8)	13 (8.9)	
DWI signal intensity, n (%)			0.091
Hyperintense	58 (90.6)	121 (82.9)	
Isointense	6 (9.4)	25 (17.1)	
TIC pattern, n (%)			0.047
Type I (persistent)	3 (4.7)	15 (10.3)	
Type II (plateau)	22 (34.4)	58 (39.7)	
Type III (washout)	39 (60.9)	73 (50.0)	
Ktrans (min^-^¹), mean ± SD	0.38 ± 0.14	0.31 ± 0.13	0.024
Kep (min^-^¹), mean ± SD	1.46 ± 0.52	1.41 ± 0.50	0.042
Ve (%), mean ± SD	0.26 ± 0.09	0.22 ± 0.08	0.015

**Figure 1:**
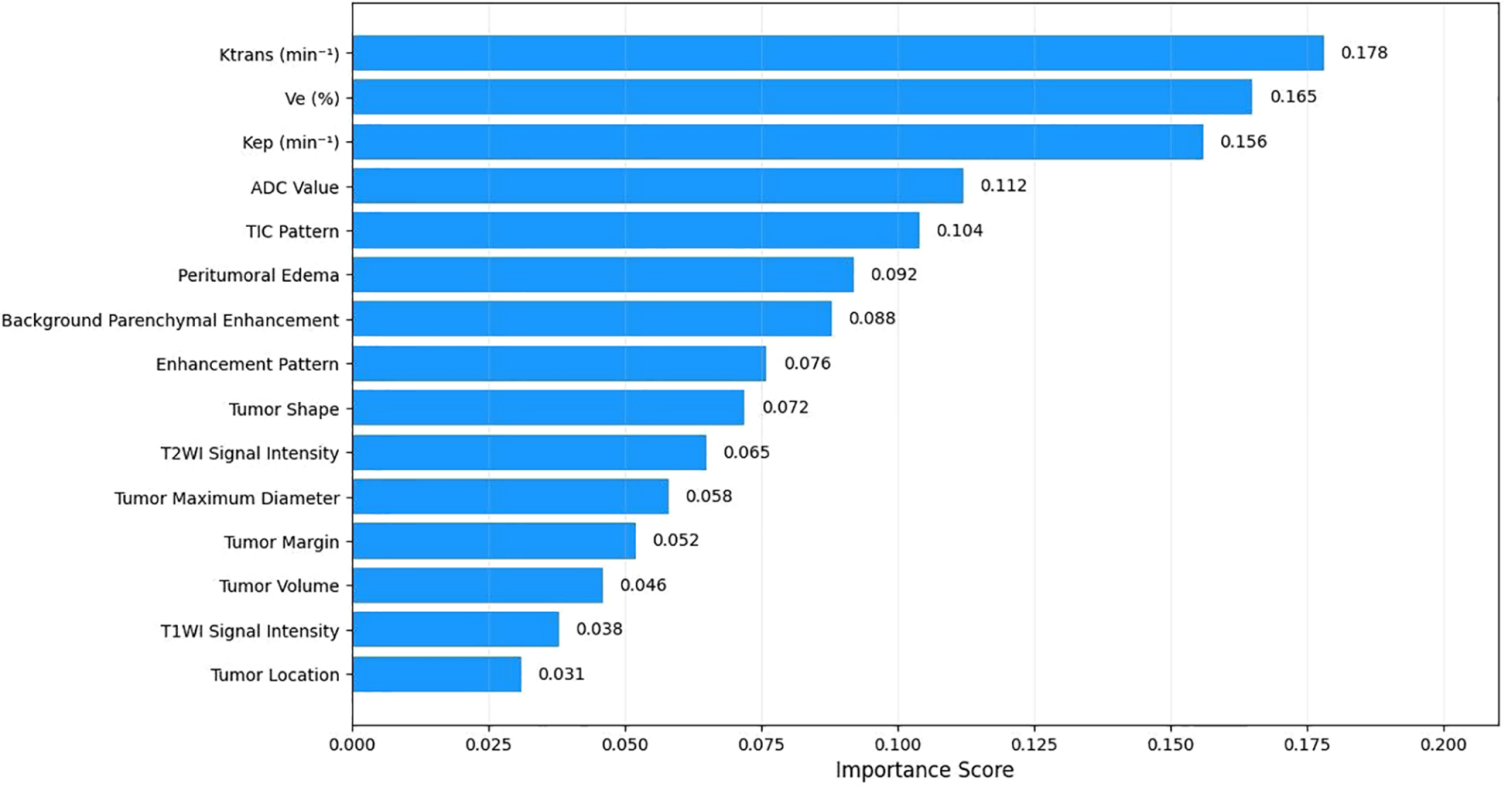
Random forest feature importance for AR status prediction. Relative importance of multi-parameter MRI features in predicting androgen receptor status in TNBC. Ktrans, Ve, Kep were identified as the most significant predictors with importance scores of 0.178, 0.165, and 0.156, respectively. Features with longer bars contribute more substantially to discriminating between AR-positive and AR-negative tumors.

In multivariate logistic regression with backward stepwise selection, the three pharmacokinetic parameters (Ktrans, Ve, Kep) emerged as independent predictors of AR status. Random forest analysis confirmed their predictive importance, with Ktrans showing the highest importance score (0.178, 95% CI: 0.135–0.221), followed by Ve (0.165, 95% CI: 0.124–0.206) and Kep (0.156, 95% CI: 0.116–0.196) (**Figure 1**).

### Radiomics feature selection

**Figure 2:**
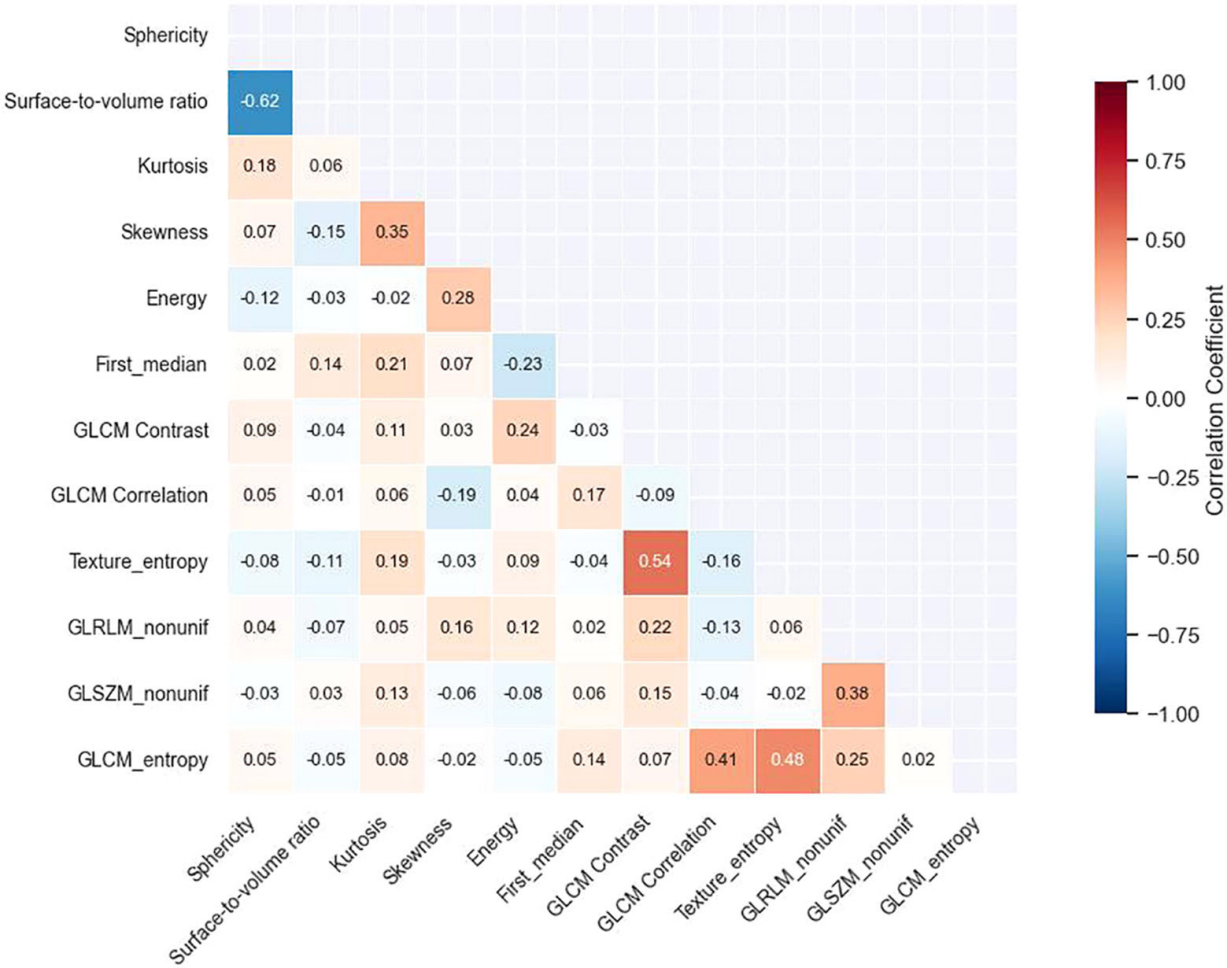
Correlation matrix of selected radiomics features. Heatmap showing Spearman correlation coefficients between selected radiomics features and AR status. Red colors indicate positive correlations while blue colors represent negative correlations, with color intensity reflecting correlation strength. The matrix demonstrates relatively low redundancy among the selected features, with most inter-feature correlations below 0.50 in absolute value.

Of the 1888 extracted radiomic features, 1706 (90.4%) demonstrated good reproducibility (ICC ≥ 0.75). Stability selection with bootstrap resampling further identified 624 stable features, which were reduced to 312 after eliminating highly correlated variables (|r| > 0.90). Application of the mRMR algorithm yielded 90 features, and the Boruta procedure ultimately identified 12 with significant discriminative value (**Supplementary Table 3**). These included shape descriptors (sphericity, surface-to-volume ratio), first-order features (kurtosis, skewness, energy), and texture features derived from GLCM (contrast, correlation), GLRLM (run-length non-uniformity), and GLSZM (size zone non-uniformity) (**Supplementary Table 4**). Correlation heatmaps confirmed low redundancy among the final selected features (**Figure 2**).

### Model performance

**Figure 3:**
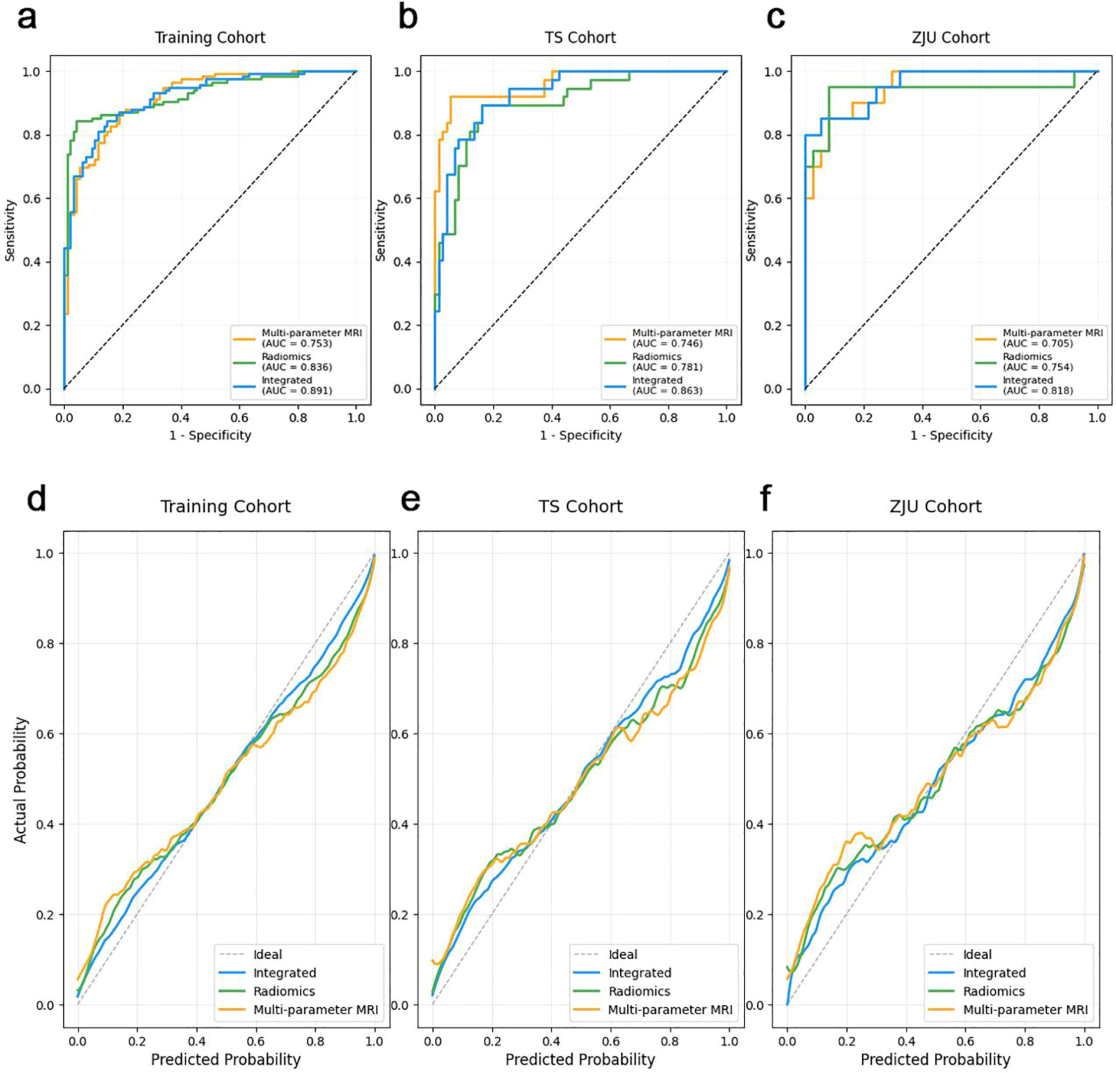
Model performance across training and validation cohorts. **(a–c)** ROC curves comparing the performance of the radiomics model, the MRI feature model, and the integrated model in the training cohort **(a)**, TS cohort **(b)**, and ZJU cohort **(c–f)** Calibration curves for the three models in the training cohort **(d)**, TS cohort **(e)**, and ZJU cohort **(f)**, showing good agreement between predicted and observed outcomes.

In the training cohort, the integrated ensemble model achieved the highest discrimination with an AUC of 0.891 (95% CI: 0.852–0.937), outperforming the radiomics model (AUC = 0.836, 95% CI: 0.782–0.884; *p* = 0.012) and the multiparametric MRI model (AUC = 0.753, 95% CI: 0.683–0.825; *p* = 0.003). At the optimal Youden index, sensitivity, specificity, and accuracy of the integrated model were 85.3% (95% CI: 68.9–95.0%), 87.2% (95% CI: 77.7–93.7%), and 86.6% (95% CI: 78.9–92.3%), respectively. External validation confirmed the superior performance of the integrated model, with AUCs of 0.863 (95% CI: 0.804–0.926) in the TS cohort and 0.818 (95% CI: 0.739–0.894) in the ZJU cohort. In both cohorts, the integrated model consistently outperformed the radiomics (AUCs = 0.781 and 0.754) and multiparametric MRI models (AUCs = 0.746 and 0.705) (**Table 4**, **Figures 3a–c**). Additional threshold-dependent metrics (accuracy, precision, F1-score, etc.) are summarized in **Supplementary Table 6**.

**Table 4:** Performance comparison of different models across three cohorts.

Model	Cohort	AUC (95% CI)	Sensitivity (95% CI)	Specificity (95% CI)	Accuracy (95% CI)	DeLong *p* value vs. Integrated Model
Radiomics	Training	0.836 (0.782-0.884)	79.4% (62.1-91.3%)	84.6% (74.7-91.8%)	82.8% (74.2-89.5%)	0.012
	TS	0.781 (0.712-0.857)	75.8% (57.7-88.9%)	83.3% (72.1-91.4%)	80.4% (71.8-87.3%)	0.018
	ZJU	0.754 (0.662-0.842)	73.9% (51.6-89.8%)	79.4% (62.1-91.3%)	77.2% (64.2-87.3%)	0.026
Multi-parameter MRI	Training	0.753 (0.683-0.825)	73.5% (55.6-87.1%)	77.9% (67.0-86.6%)	76.4% (67.5-83.9%)	0.003
	TS	0.746 (0.695-0.812)	72.7% (54.5-86.7%)	77.5% (65.8-86.7%)	75.9% (66.9-83.5%)	0.009
	ZJU	0.705 (0.624-0.783)	68.2% (45.1-86.1%)	74.5% (56.6-87.5%)	72.0% (58.3-83.1%)	0.014
Integrated	Training	0.891 (0.852-0.937)	85.3% (68.9-95.0%)	87.2% (77.7-93.7%)	86.6% (78.9-92.3%)	–
	TS	0.863 (0.804-0.926)	81.8% (64.5-93.0%)	86.4% (75.7-93.6%)	84.8% (76.8-90.9%)	–
	ZJU	0.818 (0.739-0.894)	78.3% (56.3-92.5%)	82.4% (65.5-93.2%)	80.7% (68.1-90.0%)	–

Calibration analysis demonstrated good agreement between predicted and observed outcomes. Non-significant Hosmer–Lemeshow test results across training, TS, and ZJU cohorts (all p > 0.24) indicated adequate calibration, further supported by calibration curves showing the integrated model closely aligned with the ideal reference (**Figures 3d–f**).

Cross-validation analysis in the training cohort confirmed model stability. Mean AUCs were 0.825 (SD 0.04) for the radiomics model, 0.763 (SD 0.05) for the multiparametric MRI model, and 0.881 (SD 0.03) for the integrated model. The low standard deviations reflected robust generalizability across folds.

### Model interpretation and visualization

**Figure 4:**
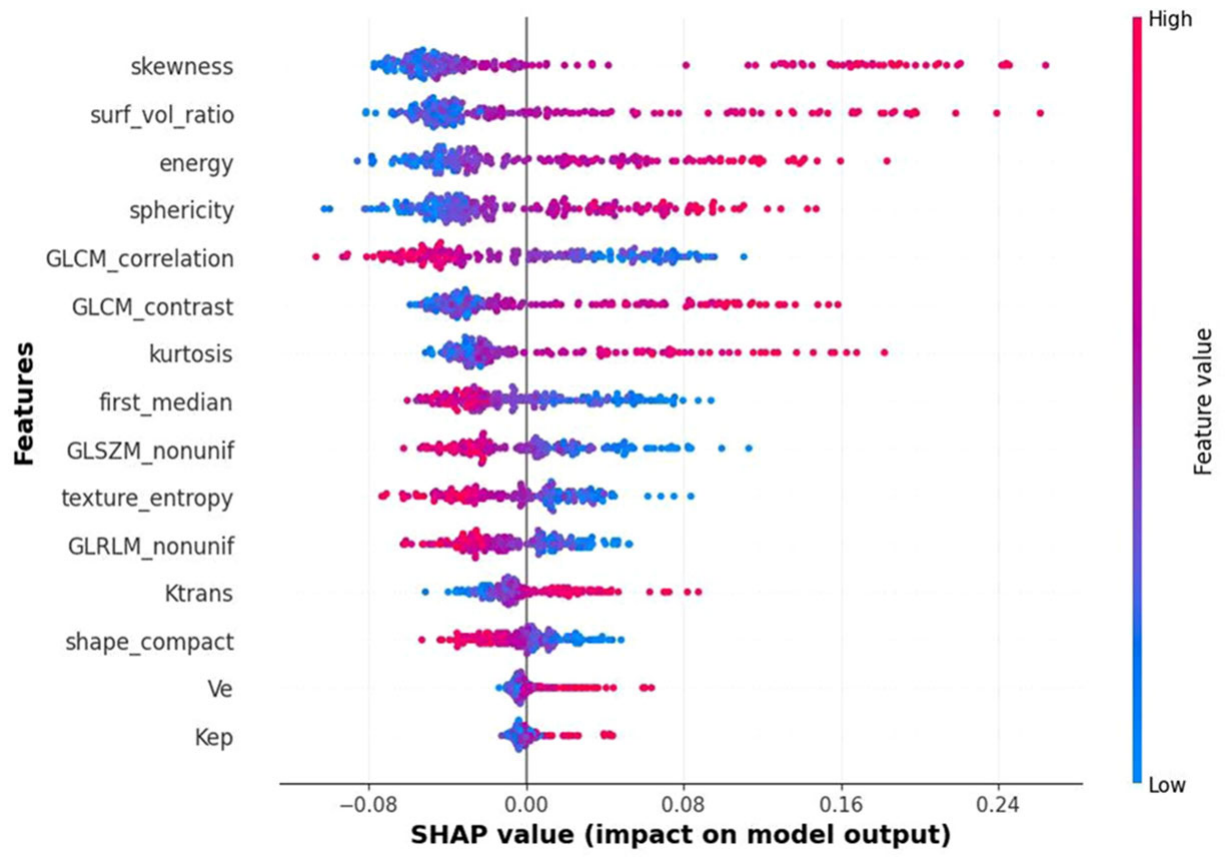
SHAP summary plot. Global feature importance for the integrated model. Features are ranked on the y-axis by overall importance. Each dot represents a SHAP value for an individual patient, with horizontal position indicating direction and magnitude of feature impact. Red denotes high feature values and blue denotes low values.

SHAP analysis provided quantitative explanations for ensemble model predictions. The summary plot (**Figure 4**) ranked features by their overall importance and directional influence on AR classification. Skewness emerged as the most influential feature: lower values consistently favored AR-positive predictions, whereas higher values were associated with AR negativity. Surface-to-volume ratio and energy ranked second and third, respectively, with higher values generally contributing to AR-negative classification. Among pharmacokinetic parameters, Ktrans showed the strongest contribution but had less impact overall compared with radiomic descriptors. Other MRI-derived features (Ve, Kep, compactness) exerted only minor influence on the final predictions.

**Figure 5:**
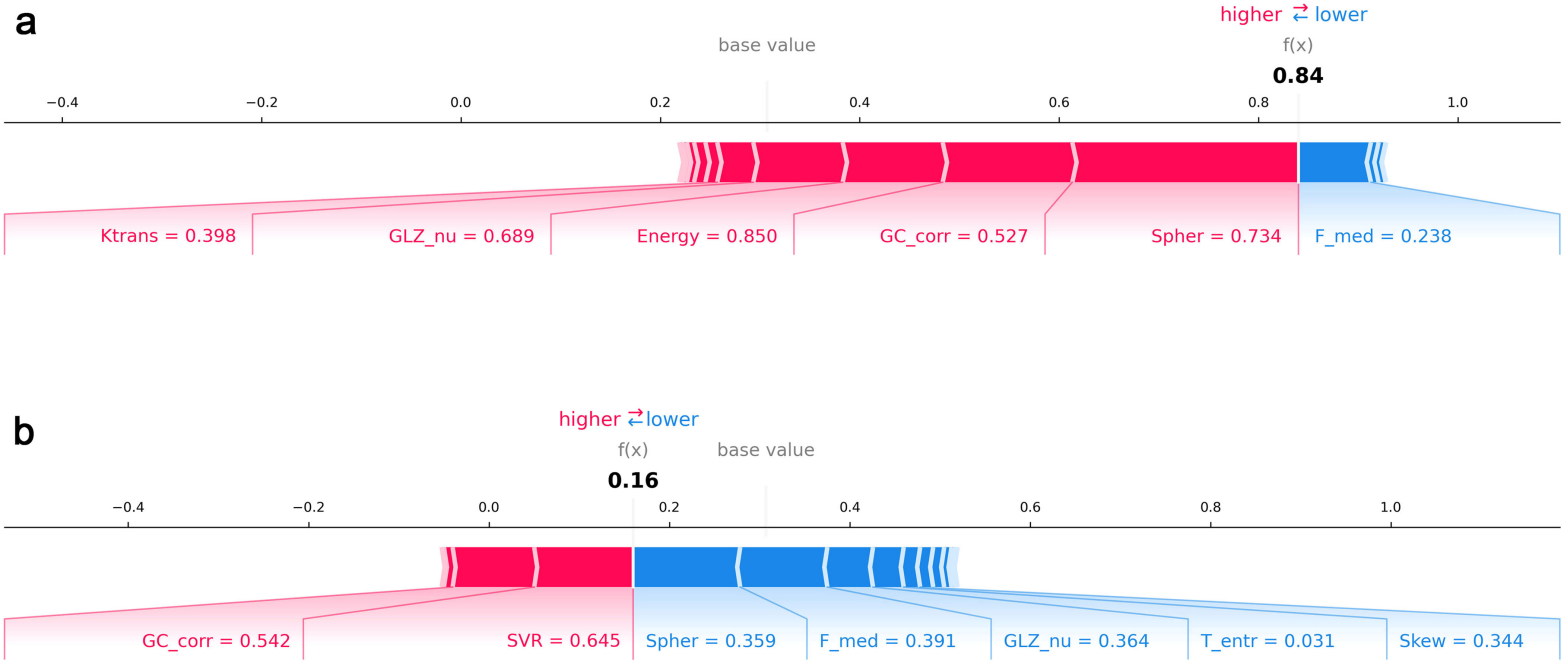
SHAP force plots for individual patient predictions. Local feature contributions for representative cases. **(a)** AR-positive patient with Ktrans, GLSZM non-uniformity, energy, GLCM correlation, and sphericity contributing positively to classification. **(b)** AR-negative patient with negative contributions from sphericity, first-order median, GLSZM non-uniformity, entropy, and skewness outweighing positive effects from GLCM correlation and surface-to-volume ratio.

Case-level explanations were visualized using SHAP force plots. In an AR-positive example (**Figure 5a**), positive contributions from Ktrans, GLSZM non-uniformity, energy, GLCM correlation, and sphericity raised the prediction probability to 0.84. In contrast, in an AR-negative case (**Figure 5b**), negative contributions from sphericity, first-order median, GLSZM non-uniformity, entropy, and skewness lowered the prediction probability to 0.16, despite limited positive influence from GLCM correlation and surface-to-volume ratio.

### Decision curve analysis

**Figure 6:**
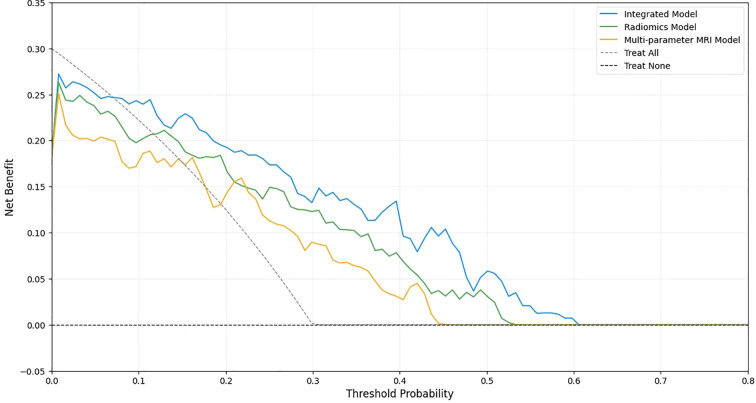
Decision curve analysis. Decision curve analysis showing net benefit across a range of threshold probabilities. The integrated model (blue) yielded the highest net benefit, followed by the radiomics (green) and multiparametric MRI (orange) models.

**Figure 7:**
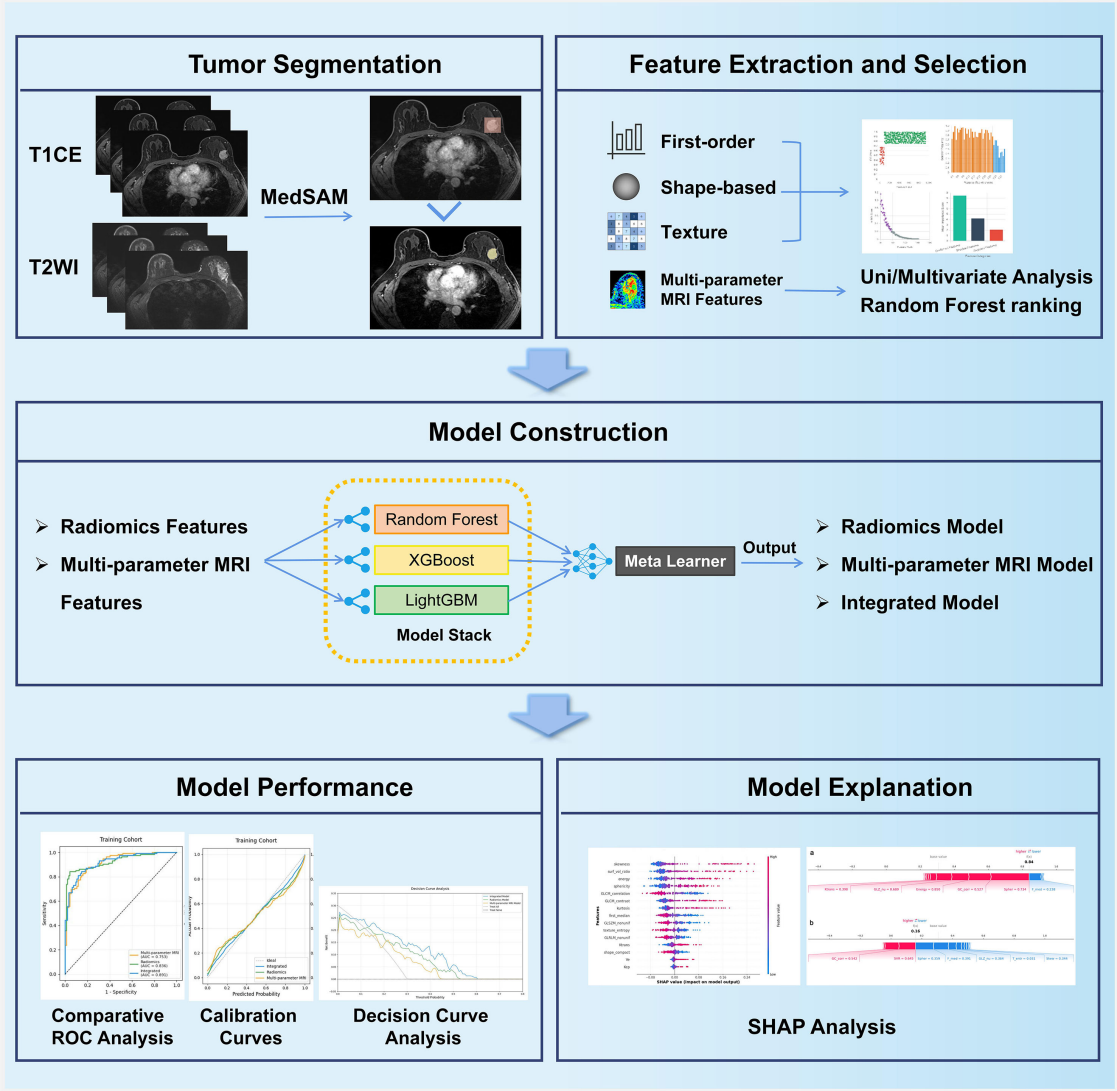
The workflow of ensemble learning model development.

Decision curve analysis (**Figure 6**) demonstrated that all three models provided net clinical benefit across a wide range of threshold probabilities (0.1–0.9) compared with treat-all or treat-none strategies. The integrated model yielded the highest net benefit, peaking at 0.58 when the threshold probability was 0.4, and maintaining superiority within the clinically relevant range of 0.2–0.8. At a threshold of 0.5, the net benefits were 0.52 for the integrated model, 0.43 for the radiomics model, and 0.35 for the multiparametric MRI model. A schematic overview of the complete study workflow is provided in **Figure 7**.

## Discussion

In this multicenter study, we developed and externally validated an interpretable ensemble imaging model integrating radiomics and multiparametric MRI for predicting AR status in TNBC. The integrated approach outperformed radiomics- or MRI-only models and maintained consistent accuracy in external validation, highlighting its potential as a noninvasive tool for patient stratification. By incorporating SHAP analysis, the model also provided transparent feature attribution, addressing interpretability concerns that have limited the clinical translation of radiomics-based methods.

Our results highlight that multiparametric MRI features reflect biologically relevant characteristics of AR-positive tumors. Pharmacokinetic parameters such as Ktrans, Ve, and Kep were significantly higher in AR-positive lesions, suggesting increased vascular permeability and extracellular space, findings consistent with previous MRI studies of tumor microenvironment (18–20). Lower ADC values indicated higher cellularity, and enhancement curve patterns further distinguished receptor status (21). The radiomic signature revealed particularly intriguing biological insights. The prominence of skewness as the top predictor suggests that AR-positive tumors exhibit asymmetric intensity distributions, potentially reflecting areas of heterogeneous cellularity and necrosis that characterize their aggressive phenotype (22). The inverse correlation between sphericity and surface-to-volume ratio (r = -0.62) indicates that AR-positive tumors demonstrate more irregular, infiltrative growth patterns compared to their AR-negative counterparts. This morphological complexity may reflect AR-mediated effects on cell adhesion and invasion pathways (23). Texture features, particularly the strong correlation between GLCM contrast and entropy (r = 0.54), captured the chaotic internal architecture of AR-positive lesions, contrast quantifying local intensity variations while entropy measuring their randomness. The GLRLM and GLSZM features provided multi-scale characterization of tissue homogeneity, from fine-grained patterns to larger zones, potentially corresponding to the distinct patterns of tumor cell clustering and stromal interaction observed in AR-positive TNBC histopathology (24). These quantitative features capture subtle tissue characteristics beyond visual assessment, with the integration of radiomics and conventional MRI providing a more comprehensive representation of tumor biology than either modality alone.

Compared with earlier work, this study provides broader validation and stronger interpretability. Xu et al. showed that texture features from DCE-MRI could distinguish AR expression (25), and Huang et al. demonstrated promising multiparametric radiomics models (26). However, these studies were single-center and lacked generalizability (27, 28). By incorporating three institutions, our study confirmed stability across independent cohorts. Notably, our implementation of medical SAM-based segmentation achieved comparable accuracy to manual delineation (Dice: 0.87 ± 0.06) while reducing segmentation time by 80%, addressing a key bottleneck in radiomics clinical translation and enhancing reproducibility across centers (29). The stacking ensemble framework, combining Random Forest, XGBoost, and LightGBM, achieved superior predictive accuracy compared with single classifiers, consistent with evidence that ensemble methods reduce overfitting and enhance robustness in radiomics analysis (30, 31). The use of SHAP further clarified the contribution of individual features, revealing that radiomic features contributed more substantially than conventional MRI parameters, which may guide future feature engineering efforts and protocol optimization (32–34).

For newly diagnosed TNBC patients, this model could provide rapid AR status assessment during initial staging MRI, potentially expediting treatment planning while awaiting immunohistochemistry results. When biopsy is limited by intratumoral heterogeneity, which causes wide variation in AR status concordance between biopsy and surgical specimens (35–38), or yields insufficient tissue for reliable staining, imaging-based prediction offers a valuable complementary assessment. Threshold probability can be tailored to clinical context: a lower threshold might be appropriate for screening patients for AR-targeted clinical trials, while a higher threshold would be suitable for definitive treatment selection. Noninvasive identification of AR-positive patients may help guide the use of AR antagonists, which have demonstrated clinical benefit in selected TNBC populations (39–42). Furthermore, by providing case-level SHAP explanatory outputs that clarify feature contributions, the model fosters greater transparency and clinical confidence compared to black-box algorithms, facilitating integration into multidisciplinary care. Decision curve analysis showed clear net benefit across relevant threshold probabilities (0.2-0.8), reinforcing the model’s value in personalized treatment decision making.

This study has limitations. Its retrospective design may introduce selection bias, and although three centers were included, the overall number of AR-positive patients was relatively small. Radiomic features were extracted from T2WI and a single post-contrast phase, which may not capture the full temporal dynamics of DCE-MRI. Pathological assessment followed ASCO/CAP guidelines, yet variability in immunohistochemistry interpretation cannot be excluded. Finally, while SHAP improved transparency, radiomic descriptors remain abstract constructs that do not always directly correspond to histopathological findings.

## Conclusions

In conclusion, we developed and externally validated an interpretable ensemble model integrating radiomics and multiparametric MRI for AR status prediction in TNBC. The model demonstrated robust and generalizable performance, with biologically meaningful features and transparent explanations. This noninvasive approach has the potential to complement biopsy, enable more reliable identification of AR-positive patients, and support personalized treatment strategies in TNBC.

## Data Availability

The original contributions presented in the study are included in the article/supplementary material. Further inquiries can be directed to the corresponding author.
