# Supplementary material for: Development and validation of an interpretable ensemble model for predicting androgen receptor status in triple-negative breast cancer: a multi-center study
Source: Front Oncol. 2026 Mar 11;16:1743315. doi: 10.3389/fonc.2026.1743315 (PMC13012927; doi:10.3389/fonc.2026.1743315)
Supplement: Supplementary file 1 [file Table1.docx]

**Supplementary Material I：MRI examination**

- **DCE-MRI image acquisition**

MRI examinations were performed with different scanners at the participating institutions (institution i and ii: Siemens Magnetom Verio 3.0T, Siemens Healthcare, Erlangen, Germany; institution iii: Philips Ingenia 3.0T, Philips Healthcare, Best, Netherlands. All institutions applied the same protocol for dynamic contrast enhancement including one pre-contrast and five dynamic post-contrast series with fat-saturated T1-weighted sequences. Contrast material (gadolinium-based agent, 0.2 mmol/kg) was injected intravenously at a rate of 2.0 mL/s, followed by a 20 mL saline flush. Contrast-enhanced images were acquired at approximately 1, 2, 3, 4, and 5 minutes after contrast injection. The detailed scanning parameters for each institution were as follows: institution i and ii: T1-weighted DCE-MRI: TR 4.51 ms, TE 1.61 ms, flip angle 10°, slice thickness 1.2 mm, FOV 340 × 340 mm, matrix 420 × 420, T2-weighted imaging: TR 8420 ms, TE 69.0 ms, slice thickness 4 mm, slice gap 1 mm, Diffusion-weighted imaging: b-value 800 s/mm², TR 10800 ms, TE 85 ms, slice thickness 4 mm, slice gap 1 mm; institution iii: T1-weighted DCE-MRI: TR 4.2 ms, TE 2.1 ms, flip angle 12°, slice thickness 1.0 mm, FOV 339 × 339 mm, matrix 407 × 407, T2-weighted imaging: TR 7890 ms, TE 74.0 ms, slice thickness 4 mm, slice gap 1 mm, Diffusion-weighted imaging: b-value 800 s/mm², TR 9700 ms, TE 89 ms, slice thickness 4 mm, slice gap 1 mm. Additional sequence parameters were harmonized during image preprocessing to minimize scanner-related variations. Image quality assurance was performed for all examinations to ensure diagnostic adequacy and comparability across institutions.

- **Pharmacokinetic Modeling Protocol**

The pharmacokinetic analysis utilized the Extended Tofts model to quantify contrast enhancement dynamics and generate voxel-based parametric maps. Individual arterial input functions (AIFs) were determined from major vessels (internal mammary artery or aorta) for each patient. Three-dimensional regions of interest (ROIs) encompassing the entire tumor volume were manually delineated on the second post-contrast phase images where tumor boundaries were optimally visualized. The ROIs were drawn to include all tumor components while maintaining a small margin from the tumor edge to minimize partial volume effects. Multiple sequential ROIs were integrated to create a comprehensive volume of interest (VOI) for three-dimensional analysis. Quality control measures included verification of adequate model fitting (R² > 0.8) and visual inspection of parametric maps for artifacts or processing errors.

**Supplementary Material II: Detailed Assessment of Multi-parameter MRI Features**

- **Morphological Features Assessment**

1. Maximum Diameter: Measured on T1-weighted contrast-enhanced sequences in three orthogonal planes (sagittal, axial, and coronal), with the largest measurement recorded.

2. Shape Assessment:

① Round/Oval: Well-circumscribed mass with round or oval configuration

② Irregular: Mass with irregular contour lacking smooth boundaries

③ Lobular: Mass with undulating contour

④ Nonmass Enhancement: Abnormal enhancement that is not a mass, distributed in a linear, segmental, regional, or diffuse pattern

3. Margin Assessment:

① Circumscribed: Well-defined margin with abrupt transition

② Irregular: Poorly defined margin without spiculations

③ Spiculated: Margin with radiating lines extending from the mass

4. Tumor Volume: Calculated after 3D segmentation from contrast-enhanced T1-weighted images

5. Tumor Location: Documented by quadrant and distance from nipple and chest wall

- **Signal Intensity Features Assessment**

1. T2-weighted Signal Intensity:

① Hyperintense: Signal intensity higher than normal fibroglandular tissue

② Isointense: Signal intensity equivalent to normal fibroglandular tissue

③ Hypointense: Signal intensity lower than normal fibroglandular tissue

2. Diffusion-weighted Signal Intensity (b=800 s/mm²):

① Hyperintense: Higher signal than surrounding breast tissue

② Isointense: Similar signal to surrounding breast tissue

③ Hypointense: Lower signal than surrounding breast tissue

3. ADC Value Measurement:

① Three ROIs (50-100 mm²) were placed on the solid portions of the tumor

② Areas of necrosis, hemorrhage, and significant artifacts were avoided

③ Measurements were taken from the ADC map

④ Mean value of the three measurements was recorded

4. Peritumoral Edema:

① Assessed on T2-weighted images

② Defined as hyperintense signal surrounding the tumor

③ Categorized as:

* Absent: No hyperintense signal outside tumor boundary

* Present: Hyperintense signal extending beyond tumor boundary

1. Background Parenchymal Enhancement (BPE):

① Assessed on the first post-contrast subtraction images

② Defined as normal fibroglandular tissue enhancement relative to pre-contrast images

③ Categorized according to BI-RADS lexicon:

* Minimal: ≤25% of fibroglandular tissue demonstrating enhancement

* Mild: 26-50% of fibroglandular tissue demonstrating enhancement

* Moderate: 51-75% of fibroglandular tissue demonstrating enhancement

* Marked: >75% of fibroglandular tissue demonstrating enhancement

④ Assessment was performed in the contralateral breast to avoid interference from the tumor

- **Enhancement Characteristics Assessment**

1. Enhancement Pattern:

① Homogeneous: Uniform enhancement throughout the tumor

② Heterogeneous: Variable enhancement within the tumor

2. Time-Intensity Curve (TIC) Analysis:

① ROIs were placed on the most enhancing portion of the tumor

② TIC patterns were classified according to the BI-RADS lexicon:

*Type I (persistent): Continuous increase of signal intensity over time

*Type II (plateau): Initial increase followed by flattening of the enhancement curve

*Type III (washout): Initial increase followed by decrease of signal intensity

3. Initial Enhancement Slope:

① Calculated as: (SI₁ - SI₀)/(SI₀ × T₁) × 100%

② Where SI₁ is the signal intensity on the first post-contrast image, SI₀ is the signal intensity on the pre-contrast image, and T₁ is the time in minutes between the pre-contrast and first post-contrast acquisitions

③ Measured in percentage increase per minute

- **Pharmacokinetic Parameters Assessment**

1. Ktrans (volume transfer constant): representing contrast agent flux from plasma to extravascular extracellular space (EES), measured in min^-1^

2. Kep (rate constant): indicating contrast agent reflux from EES to plasma, measured in min^-1^

3. Ve (EES volume fraction): quantifying the proportional volume of extravascular extracellular space relative to total tissue volume, expressed as a percentage

- **MRI Feature Selection Process**

1. Univariate analysis was performed to identify features significantly associated with AR status

2. Features with p < 0.05 were entered into multivariate logistic regression with backward stepwise selection

3. Entry threshold: p < 0.05, removal threshold: p > 0.10

4. Random forest model was built using selected features to calculate importance scores

5. Feature importance was measured by mean decrease in Gini impurity

6. 95% confidence intervals for importance scores were generated using bootstrap resampling

**Supplementary Material III: Detailed Radiomics Feature Selection and Model Development**

**Feature Selection Process**

Our feature selection framework followed a multi-step approach designed to identify the most robust and discriminative features while reducing redundancy and overfitting risk. The complete process is described below:

**Step 1: Reproducibility Assessment**

1. All 1888 initially extracted features were evaluated for reproducibility using intraclass correlation coefficient (ICC) based on repeated segmentations

2. Features with ICC values < 0.75 were excluded

3. Remaining features: 1706 (90.4% of initial features)

**Step 2: Stability Selection**

1. Bootstrap resampling with 1000 iterations was performed

2. For each iteration, 80% of the samples were randomly selected

3. Features were ranked by importance in each bootstrap sample using random forest

4. Features appearing in the top 20% of importance ranking in at least 70% of iterations were retained

5. Remaining features: 624 (36.6% of reproducible features)

**Step 3: Correlation Analysis**

1. Spearman's rank correlation coefficients were calculated between all pairs of features

2. For highly correlated feature pairs (|r| > 0.90), the feature with higher ICC value was retained

3. This step reduced redundancy while maintaining reproducibility

4. Remaining features: 312 (50.0% of stable features)

**Step 4: Minimum Redundancy Maximum Relevance (mRMR)**

1. Features were ranked according to the mRMR criterion

2. The mutual information difference scheme was employed:

Score(f) = I(f;c) - 1/|S| Σ I(f;f_i), where:

* I(f;c) = mutual information between feature f and class label c

* I(f;f_i) = mutual information between feature f and selected feature f_i

* |S| = number of already selected features

3. Top 90 features with highest mRMR scores were retained

4. Remaining features: 90 (28.8% of non-redundant features)

**Step 5: Boruta Algorithm**

1. Random forest-based all-relevant feature selection

2. Shadow features were created by permuting original features

3. Features with importance consistently higher than their shadows were retained

4. Significance was assessed over 100 independent iterations

5. Remaining features: 12 (13.3% of mRMR features)

**Step 6: Statistical Verification**

1. Mann-Whitney U test was performed for each selected feature

2. Benjamini-Hochberg procedure was applied to control the false discovery rate

3. All 12 features showed significant difference between AR-positive and AR-negative groups (p < 0.05 after correction)

4. Final selected features: 12

- **Model Development**

Three prediction models were constructed using the same ensemble learning approach:

**1. Radiomics Model**

① Input: 12 selected radiomics features

② Architecture:

* Base learners: Random Forest, XGBoost, LightGBM

* Meta-learner: Logistic Regression with L2 regularization

**2. Multi-parameter MRI Model**

① Input: 3 selected MRI parameters (Ktrans, Ve, Kep)

② Architecture: Same as radiomics model

**3. Integrated Model**

① Input: Combined 12 radiomics features and 3 MRI parameters

② Architecture: Same as radiomics model

- **Ensemble Learning Configuration**

**Base Learners:**

**1. Random Forest:**

- n_estimators: 100

- max_depth: 6

- min_samples_leaf: 5

- class_weight: 'balanced'

- criterion: 'gini'

- random_state: 42

**2. XGBoost:**

- n_estimators: 100

- learning_rate: 0.01

- max_depth: 6

- subsample: 0.8

- colsample_bytree: 0.8

- reg_alpha: 0.1 (L1 regularization)

- reg_lambda: 0.1 (L2 regularization)

- scale_pos_weight: 2.28 (to handle class imbalance)

- random_state: 42

**3. LightGBM:**

- n_estimators: 100

- learning_rate: 0.01

- num_leaves: 31

- max_depth: 6

- feature_fraction: 0.8

- bagging_fraction: 0.8

- reg_alpha: 0.1

- reg_lambda: 0.1

- random_state: 42

**Meta-learner:**

- Logistic Regression:

- C: 1.0 (inverse of regularization strength)

- penalty: 'l2'

- solver: 'liblinear'

- class_weight: 'balanced'

- random_state: 42

**Cross-validation Strategy:**

- Nested 5-fold cross-validation

- Outer loop: Evaluating overall model performance

- Inner loop: Generating meta-features for meta-learner training

- Stratified sampling to maintain class distribution

**Feature Importance Calculation:**

- SHAP (SHapley Additive exPlanations) values were calculated for each feature

- Base learner importances were combined using weighted averaging

- Weights were determined by the performance of each base learner

**Hyperparameter Optimization:**

- Grid search with cross-validation was used to optimize hyperparameters

- Objective function: Maximize AUC

- Search space:

* Random Forest: max_depth [3, 6, 9], min_samples_leaf [1, 3, 5]

* XGBoost: learning_rate [0.01, 0.05, 0.1], max_depth [3, 6, 9]

* LightGBM: learning_rate [0.01, 0.05, 0.1], num_leaves [20, 31, 50]

* Meta-learner: C [0.1, 1.0, 10.0]

**Supplementary Material IV：Supplementary Tables**

**Table S1. Performance Metrics of MedSAM-assisted Segmentation**

| **Metric** | **MedSAM vs Manual** | **Inter-observer (Manual)** | **Inter-observer (MedSAM)** | ***p* value*** |
| --- | --- | --- | --- | --- |
| **Volumetric Overlap** |  |  |  |  |
| Dice Similarity Coefficient | 0.87 ± 0.06 | 0.81 ± 0.08 | 0.89 ± 0.05 | <0.001 |
| Jaccard Index | 0.77 ± 0.07 | 0.69 ± 0.11 | 0.80 ± 0.06 | <0.001 |
| **Surface Distance (mm)** |  |  |  |  |
| Hausdorff Distance | 4.8 ± 1.7 | 6.2 ± 2.4 | 4.3 ± 1.5 | <0.001 |
| 95% Hausdorff Distance | 2.9 ± 0.9 | 3.8 ± 1.2 | 2.7 ± 0.8 | <0.001 |
| Average Surface Distance | 1.1 ± 0.4 | 1.6 ± 0.7 | 0.9 ± 0.4 | <0.001 |
| **Volumetric Measurements** |  |  |  |  |
| Volume Difference (%) | 7.6 ± 5.3 | 12.8 ± 9.4 | 6.2 ± 4.1 | <0.001 |
| **Reproducibility (ICC)** |  |  |  |  |
| Tumor Volume | 0.92 (0.88-0.95) | 0.82 (0.75-0.89) | 0.91 (0.87-0.95) | 0.003 |
| Maximum Diameter | 0.94 (0.90-0.96) | 0.85 (0.78-0.90) | 0.93 (0.89-0.96) | 0.002 |
| **Time Efficiency (min)** | 2.8 ± 0.7 | 15.3 ± 3.2 | 2.8 ± 0.7 | <0.001 |

**Table S2. ICC Values for Selected Radiomics Features Extracted from Manual vs. MedSAM-assisted Segmentation**

| **Feature Category** | **Feature Name** | **Sequence** | **Manual vs. MedSAM** | **Inter-observer (Manual)** | **Inter-observer (MedSAM)** | **Feature Stability** |
| --- | --- | --- | --- | --- | --- | --- |
|  |  |  | ICC  (95%CI) | ICC  (95%CI) | ICC  (95%CI) | CV (%) |
| **Shape-based** |  |  |  |  |  |  |
|  | Sphericity | T2WI | 0.89 (0.84-0.93) | 0.86 (0.79-0.91) | 0.91 (0.86-0.95) | 4.2 |
|  | Surface-to-volume ratio | T2WI | 0.86 (0.80-0.91) | 0.84 (0.77-0.89) | 0.89 (0.83-0.93) | 5.8 |
| **First-order** |  |  |  |  |  |  |
|  | Kurtosis | T2WI | 0.90 (0.85-0.94) | 0.88 (0.82-0.92) | 0.93 (0.89-0.96) | 6.1 |
|  | Skewness | DCE-MRI | 0.88 (0.82-0.92) | 0.87 (0.81-0.91) | 0.90 (0.85-0.94) | 7.3 |
|  | Energy | DCE-MRI | 0.91 (0.86-0.95) | 0.89 (0.84-0.93) | 0.93 (0.88-0.96) | 5.4 |
|  | First_median | T2WI | 0.87 (0.81-0.91) | 0.85 (0.78-0.90) | 0.89 (0.84-0.93) | 6.7 |
| **Texture** |  |  |  |  |  |  |
|  | GLCM Contrast | DCE-MRI | 0.88 (0.82-0.92) | 0.85 (0.78-0.90) | 0.92 (0.87-0.95) | 8.9 |
|  | GLCM Correlation | T2WI | 0.85 (0.78-0.90) | 0.81 (0.73-0.87) | 0.89 (0.84-0.93) | 9.2 |
|  | Texture_entropy | DCE-MRI | 0.86 (0.80-0.91) | 0.83 (0.76-0.88) | 0.90 (0.85-0.94) | 8.1 |
|  | GLRLM_nonunif | DCE-MRI | 0.86 (0.80-0.91) | 0.83 (0.76-0.88) | 0.89 (0.84-0.93) | 9.5 |
|  | GLSZM_nonunif | DCE-MRI | 0.83 (0.76-0.88) | 0.79 (0.71-0.85) | 0.87 (0.81-0.91) | 10.3 |
|  | GLCM_entropy | DCE-MRI | 0.82 (0.75-0.87) | 0.78 (0.70-0.84) | 0.86 (0.80-0.91) | 11.1 |

**Table S3. Feature Selection Results at Each Step**

| **Selection Step** | **Features Retained** | **Percentage of Previous Step** | **Key Features Added/Removed** |
| --- | --- | --- | --- |
| Initial features | 1888 | 100% | All extracted radiomic features |
| ICC filtering (≥0.75) | 1706 | 90.4% | Removed unstable features (primarily wavelet features) |
| Stability selection | 624 | 36.6% | Retained frequently high-ranked features across bootstrap samples |
| Correlation analysis | 312 | 50.0% | Removed highly redundant features (\|r\| > 0.90) |
| mRMR | 90 | 28.8% | Optimized relevance-redundancy trade-off |
| Boruta algorithm | 12 | 13.3% | Identified statistically significant features |
| Final set | 12 | 100% | Shape (2), First-order (3), Texture (7) |

**Table S4. Selected Radiomic Features: Statistical Comparison Between AR-positive and AR-negative Groups**

| **Feature** | **AR-positive (n=64)** | **AR-negative (n=146)** | ***p* value*** | **AUC**  **(95% CI)** | **Importance Score** |
| --- | --- | --- | --- | --- | --- |
| **Shape-based Features** |  |  |  |  |  |
| Sphericity | 0.63 ± 0.12 | 0.78 ± 0.09 | <0.001 | 0.81 (0.76-0.86) | 0.82 |
| Surface-to-  volume ratio | 0.28 ± 0.05 | 0.21 ± 0.04 | <0.001 | 0.79 (0.73-0.84) | 0.73 |
| **First-order** |  |  |  |  |  |
| Kurtosis | 4.32 ± 1.18 | 2.87 ± 0.93 | <0.001 | 0.80 (0.75-0.85) | 0.75 |
| Skewness | 0.86 ± 0.29 | 0.52 ± 0.24 | <0.001 | 0.78 (0.73-0.83) | 0.70 |
| Energy | 98.6 ± 24.7 | 76.3 ± 19.4 | <0.001 | 0.77 (0.71-0.82) | 0.68 |
| First_median | 127.3 ± 31.5 | 148.7 ± 28.2 | <0.001 | 0.75 (0.69-0.80) | 0.61 |
| **Texture** |  |  |  |  |  |
| GLCM Contrast | 24.8 ± 6.3 | 18.1 ± 5.1 | <0.001 | 0.81 (0.75-0.86) | 0.78 |
| GLCM Correlation | 0.42 ± 0.11 | 0.56 ± 0.13 | <0.001 | 0.77 (0.71-0.82) | 0.67 |
| Texture_entropy | 5.89 ± 0.92 | 4.76 ± 0.85 | <0.001 | 0.76 (0.70-0.81) | 0.62 |
| GLRLM_nonunif | 142.3 ± 38.5 | 103.6 ± 30.2 | <0.001 | 0.78 (0.73-0.83) | 0.71 |
| GLSZM_nonunif | 68.4 ± 17.5 | 52.1 ± 14.3 | <0.001 | 0.76 (0.70-0.81) | 0.65 |
| GLCM_entropy | 4.87 ± 0.74 | 4.12 ± 0.65 | <0.001 | 0.76 (0.70-0.81) | 0.63 |

**Table S5. Hyperparameter Optimization Results for Ensemble Architecture**

| **Model** | **Parameter** | **Values Tested** | **Optimal Value** | **CV Performance (AUC)** |
| --- | --- | --- | --- | --- |
| **Random Forest** | max_depth | 3, 6, 9 | 6 | 0.805 |
|  | min_samples_leaf | 1, 3, 5 | 5 | 0.798 |
|  | n_estimators | 50, 100, 200 | 100 | 0.802 |
|  | class_weight | none, 'balanced' | 'balanced' | 0.805 |
| **XGBoost** | learning_rate | 0.01, 0.05, 0.1 | 0.01 | 0.818 |
|  | max_depth | 3, 6, 9 | 6 | 0.815 |
|  | subsample | 0.6, 0.8, 1.0 | 0.8 | 0.810 |
|  | colsample_bytree |  |  |  |
|  | reg_alpha |  |  |  |
|  | reg_lambda |  |  |  |
| **LightGBM** | learning_rate | 0.01, 0.05, 0.1 | 0.01 | 0.812 |
|  | num_leaves | 20, 31, 50 | 31 | 0.809 |
|  | feature_fraction | 0.6, 0.8, 1.0 | 0.8 | 0.807 |
|  | bagging_fraction |  |  |  |
|  | reg_alpha |  |  |  |
|  | reg_lambda |  |  |  |
| **Meta-learner** | C | 0.1, 1.0, 10.0 | 1.0 | 0.836 |
| **(Logistic Regression)** | penalty | 'l1', 'l2' | 'l2' | 0.834 |
|  | solver | 'liblinear', 'lbfgs' | 'liblinear' | 0.836 |
|  | class_weight | none, 'balanced' | 'balanced' | 0.836 |

**Table S6. Threshold-dependent metrics for each model and cohort**

| **Cohort** | **Model** | **Threshold**  **(Youden index)** | **Accuracy** | **Precision** | **Sensitivity** | **Specificity** | **F1 score** | **Balanced Accuracy** |
| --- | --- | --- | --- | --- | --- | --- | --- | --- |
| Training cohort | Radiomics | 0.47 | 0.83 | 0.70 | 0.84 | 0.84 | 0.76 | 0.84 |
|  | MRI | 0.45 | 0.76 | 0.61 | 0.78 | 0.78 | 0.69 | 0.78 |
|  | Integrated | 0.49 | 0.87 | 0.74 | 0.91 | 0.86 | 0.82 | 0.88 |
| TS cohort | Radiomics | 0.46 | 0.80 | 0.64 | 0.89 | 0.83 | 0.74 | 0.86 |
|  | MRI | 0.45 | 0.76 | 0.59 | 0.82 | 0.81 | 0.69 | 0.82 |
|  | Integrated | 0.48 | 0.85 | 0.70 | 0.93 | 0.87 | 0.80 | 0.90 |
| ZJU cohort | Radiomics | 0.47 | 0.77 | 0.55 | 0.79 | 0.79 | 0.65 | 0.79 |
|  | MRI | 0.46 | 0.72 | 0.50 | 0.71 | 0.77 | 0.59 | 0.74 |
|  | Integrated | 0.48 | 0.81 | 0.63 | 0.86 | 0.84 | 0.73 | 0.85 |
